# Relation between Crystal Structures of Precursors and Final Products: Example of Vitamin D Intermediates

**DOI:** 10.3390/molecules25081802

**Published:** 2020-04-14

**Authors:** Monika Wanat, Maura Malinska, Andrzej Kutner, Krzysztof Woźniak

**Affiliations:** 1 Biological and Chemical Research Centre, Department of Chemistry, University of Warsaw, 101 Żwirki i Wigury, 02-089 Warszawa, Poland; mwanat@uw.edu.pl (M.W.); mmalinska@chem.uw.edu.pl (M.M.); 2 College of Inter-Faculty Individual Studies in Mathematics and Natural Sciences (MISMaP), University of Warsaw, 2C Stefana Banacha, 02-097 Warszawa, Poland; 3 Faculty of Pharmacy, Department of Bioanalysis and Drug Analysis, Medical University of Warsaw, 1 Stefana Banacha, 02-097 Warszawa, Poland; andrzej.kutner@wum.edu.pl

**Keywords:** hydrogen bonds, energy frameworks, intermolecular interactions

## Abstract

In this paper, we proved that the solid-state structure of vitamin D analog is well represented by the structures of its structural fragments. This is important in predicting the biological activity of vitamin D analogs that are not available in the solid form. The previously published crystal structure of advanced vitamin D intermediate provided additional insights into vitamin D properties. A similar analysis based on simple vitamin D intermediate analogues showed that precursors crystallized in the space groups typical for vitamins D; geometrical parameters were related to the corresponding parameters in the vitamin D analogues; and crystal structures of the basic intermediates and their final products contained similar intermolecular interactions, essential for the infinite hydrogen bond motif observed in the vitamin D analogues. The energy of these interactions is related as shown by theoretical calculations, that is, energy frameworks analysis. Moreover, analysis of the hydrogen bonds motifs revealed a relation between these motifs and the absolute configuration of basic intermediates as well as the space orientation of the exocyclic methylene group in the final structures.

## 1. Introduction

**Figure 1:**
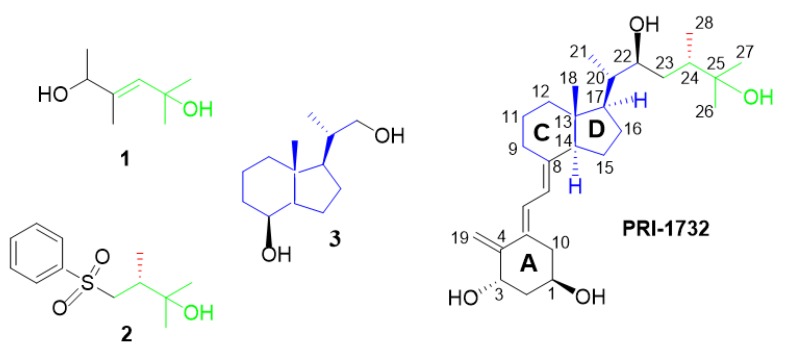
Structures of the vitamin D intermediates **1**, **2**, and **3**. The respective structural fragments of vitamin D are marked by colour and presented as an example final product (PRI-1732) with the distinctive numbering system of the vitamins D.

Vitamins D belong to a group of fat-soluble secosteroids called calciferols. The two major forms of vitamin D are ergocalciferol (vitamin D_2_) and cholecalciferol (vitamin D_3_). Both forms are activated by the hydroxylations at C-1 and C-25 carbons (Figure 1). Most of the vitamin D biological functions of vitamin D are mediated through the vitamin D receptor (VDR), which is a part of the nuclear receptor family [1,2,3,4,5,6,7]. The VDR is present in and regulates cells of the immune, respiratory, circulatory, muscle, and other systems [4,8,9,10]. The biological activity of these compounds is broad. For instance, vitamin D inhibits the proliferation of cancer cell as well as increases the expression of DNA repairing factors [11]. Additionally, vitamin D regulates calcium and phosphate homeostasis. Vitamin D and their analogues are broadly used for the treatment of several diseases. For instance, calcipotriol [12] and tacalcitol [13] are used against psoriasis, whereas paricalcitol [14] is used against secondary hyperparathyroidism. It is well documented that the biological and clinical importance has the entire vitamin D molecule consisting of four subfragments. In other words, the analogue of vitamin D shows biological activity only when it contains all four structural parts: di-substituted A-ring, oligo-ene linker, CD-ring system, and side-chain bearing the terminal hydroxyl (Figure 1). None of these structural fragments separately showed any significant activity. The same is true in the case of the advanced vitamin D intermediates we studied previously, as they usually have their hydroxyls protected and thus are deprived of the possibility of hydrogen bond formation. However, the simple structural fragments having their X-ray structures solved might be a valuable source of data in predicting the solid-state structure of the final vitamin D analogues. Despite broad applications and interests in the chemistry of vitamin D, less than 20 crystal structures of active metabolites and various analogues of vitamin D have been solved and deposited in the Cambridge Structural Database (CSD) [15,16,17,18], while ca. 20, 20, and 40 structures of widely used other pharmaceutical compounds were deposited, that is, ibuprofen, acetylsalicylic acid, and acetaminophen, respectively [19,20]. This is because of the challenges present in crystallization and X-ray studies of poor-quality vitamin D analogue crystals. The low ability of crystallization of vitamin D analogues may arise from the relatively large size of the vitamin D molecule and most likely from its high flexibility. In a vitamin D structure, both rigid parts, the cyclohexane A-ring and indane CD-ring system (Figure 1, PRI-1732), are connected by a triene system that adopts a number of geometrical conformations. Additionally, the structure contains a very flexible aliphatic side-chain that might exist in an endless number of conformations, only slightly limited by a long distance hydrogen bonding with the A-ring hydroxyls.

Until now, several crystal structures of the inactive forms of vitamin D analogues have been published [21,22,23,24,25,26,27,28], as well as very few structures of the active metabolites and analogues [22,23,27]. Recently, we solved the structure of the advanced intermediate of vitamin D called BNR-1 [16]. These analogues were used in conformational space studies of the vitamin D analogues in their crystal environments. These studies revealed that nine types of hydrogen bonds (marked as HB1–HB9, Figure S7) contributed to the lattice energy. These hydrogen bonds may form hydrogen bond motifs, that is, infinite and discrete hydrogen bond chains or R_4_^4^ and R_6_^6^ hydrogen bond rings [29]. These patterns are strongly related to the position of the exocyclic methylene group, as this group stabilizes hydrophobic interactions with a triene system only in the natural position, that is, attached to the C10 carbon atom. We showed that infinite hydrogen bond chains are essential in the interactions of vitamin D analogues and lead to a chair β-conformation that has lower energy than the α-conformation, and thus enables strong interactions with the VDR [16].

The advanced intermediate BNR crystallized in the P2_1_2_1_2_1_ space group—typical for vitamin D analogues. The values of the geometrical parameters of the A-ring in this compound were between the values of these analogues that adopted either the α- or β-conformations. In this work, we present a similar analysis, however, focused on less advanced intermediates used as starting building blocks of the vitamin D analogues. This analysis explains the relation between crystal structures of the final products and their intermediates, which may be useful as, often, obtaining the crystals of vitamins D analogues is impossible. Therefore, the structural analysis of precursors may provide additional information despite the limits arising from the ability of vitamins D to crystallize. Structural fragments **1** and **2** represent modified side chains precursors. This part of vitamins D is often chemically modified and, owing to the flexibility of this side chain, it significantly influences the binding affinity of the analogues with VDR [30]. For instance, replacing either the C26- and C27-methyl groups with ethyl moieties resulted in the superagonist, KH1060, which binds to VDR with high affinity as well as decreased calcemic side effects [28]. Inhoffen–Lythgoe diol as structural precursor (**3**) is modified at the rigid CD-ring system, which is less often modified [31]. Diol **3** is obtained by reductive work-up of the ozonolysis product of vitamin D_2_ and is widely used [32] for the semi-total synthesis of vitamin D analogues. Although Inhoffen–Lythgoe diol (**3**) has been widely used as the main structural fragment in the synthesis of vitamin D analogues over the years, its crystal structure was not solved until now. However, crystal structures of analogues of diol **3**, mostly with the protecting group, are available in CSD (CSD refcodes: CINZEE, CINZUU [33], ESIJAR [34], GILXEG [35], SEVSAP [36]). Here, we present a discussion on the geometrical and energetic aspects of the hydrogen bonds of vitamin D analogues and their intermediates.

## 2. Results and Discussion

### 2.1. X-ray Diffraction Studies

**Figure 2:**
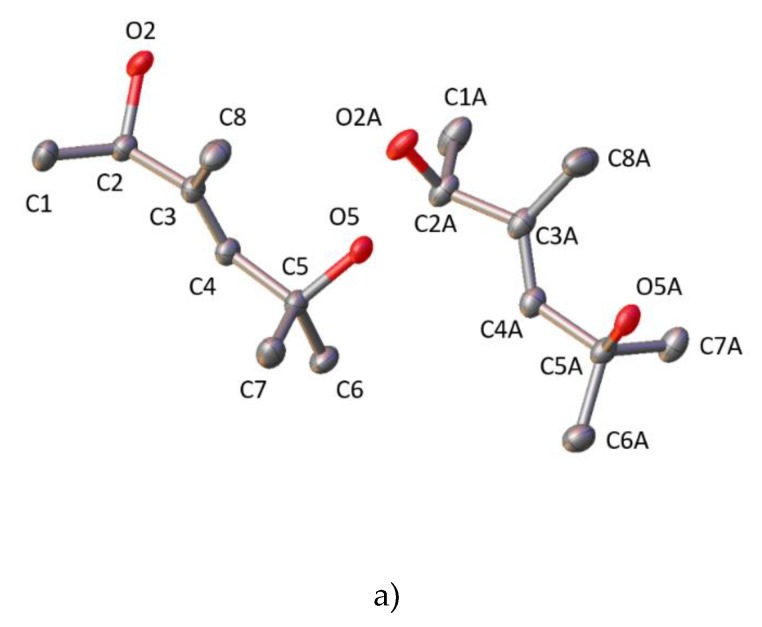

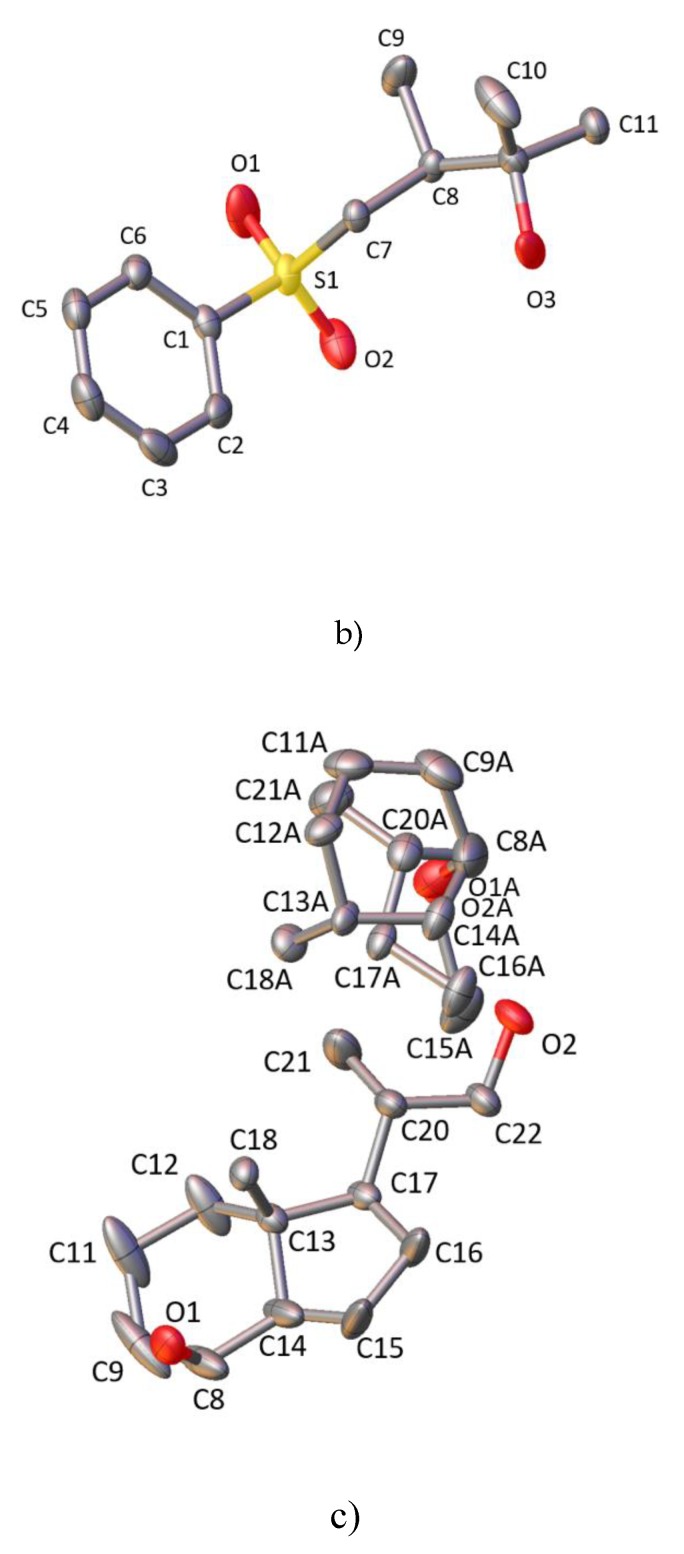
Displacement ellipsoid plot (50% probability level) of (**a**) **1**, (**b**) **2**, and (**c**) **3**. The hydrogen atoms were omitted for clarity.

**Figure 3:**
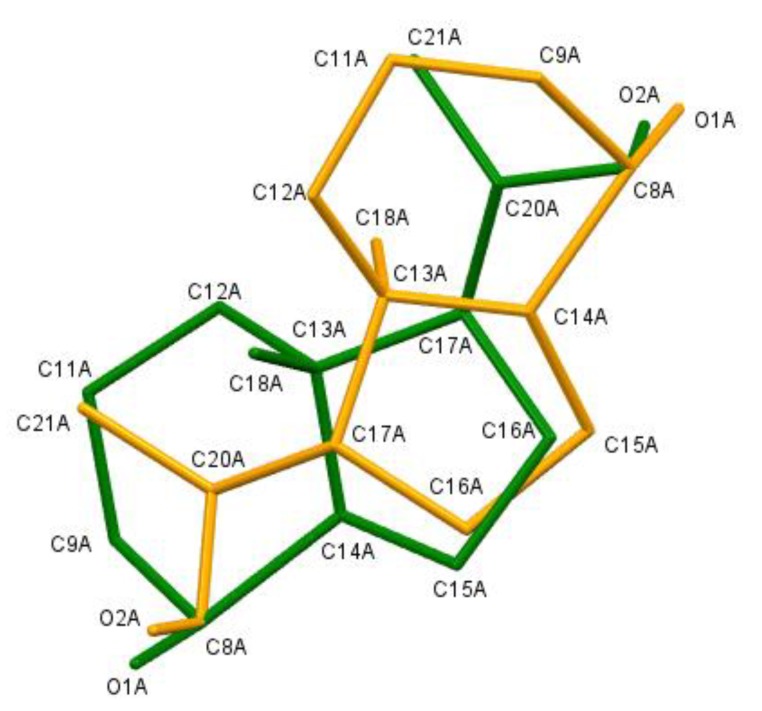
Disorder part of structure **3**. Atoms marked as green and orange have occupancy equal to 0.5. Atom C8A has occupancy equal to 1 and is present in both the green and orange molecules. The hydrogen atoms were omitted for clarity.

Here, we report the crystal structures of three precursors—**1**, **2**, and **3** (Figure 1, Figures S1–S3)—used in the synthesis of the vitamin D analogues. Diol **1** (Figure 2a) crystallizes in the P2_1_/c space group with two molecules in the asymmetric part of the unit cell. These two molecules are a pair of enantiomers (Figure 3) forming a racemic crystal. Sulfone **2** (Figure 2b) crystallizes in the P2_1_2_1_2_1_ space group with one molecule in the asymmetric unit. Diol **3** (Figure 2c) crystallizes in the C2 space group. Two molecules are present in the asymmetric unit, and one of the molecules is disordered around the 2-fold axis (Figure 3, Figure S4, 3D illustrations are available in additional materials).

### 2.2. Analogues of Vitamin D

Compounds of **1**, **2**, and **3** might be used for chemical synthesis of the vitamin D analogues, PRI-1730, PRI-1731, and PRI-1732, which were described in our previous studies [16,37]. Analogues PRI-1730, PRI-1731, and PRI-1732 crystallize in the P2_1_2_1_2_1_, C2, and P2_1_2_1_2 space groups, respectively. Many vitamin D analogues that contain unsaturated bonds in their structures crystallize in the monoclinic crystal system, whereas analogues with additional hydroxyl groups mostly crystallize in the orthorhombic crystal system. Although it is not a general rule, this could explain observations that **1** and **3** crystallize in monoclinic crystal systems and that **2** crystallizes in the orthorhombic crystal system. Nonetheless, all of these compounds crystallize in the space groups that are also typical for the vitamins D.

### 2.3. Geometry Analysis

In order to compare the geometry of the final product and substrate, we analysed the differences between the corresponding bonds of the vitamin D analogues and structures **1**, **2**, and **3** (Table 1 and Table 2). For instance, in **1**, the bond corresponding to C24-C25 was C5A-C4A (Figure 1). The analysis revealed that, in general, the differences between bond lengths are small and no higher than 0.041 Å. Comparison of the C-C bonds from the side chains resulted in differences lower than 0.018Å for the methyl group (i.e., bonds C25-C26 and C25-C27) and larger than 0.013Å for C24-C25 bond. A higher agreement was observed for the C-O bonds as the differences were smaller than 0.011Å. Moreover, comparison of the bond lengths of the CD-ring system of the vitamin D analogues and **3** revealed differences smaller than 0.028Å. Geometry analysis showed that not only the rigid part of the substrates had geometry parameters similar to the final structure. Further, the flexible parts were similar despite the presence of the leaving group in the structure of **2**.

### 2.4. Intermolecular Interactions

#### 2.4.1. Hydrogen Bonds Motifs

**Figure 4:**
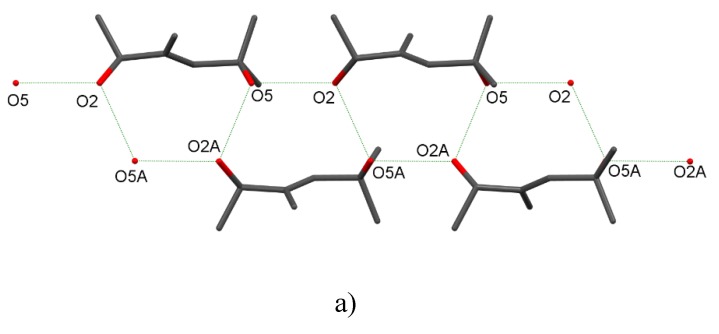

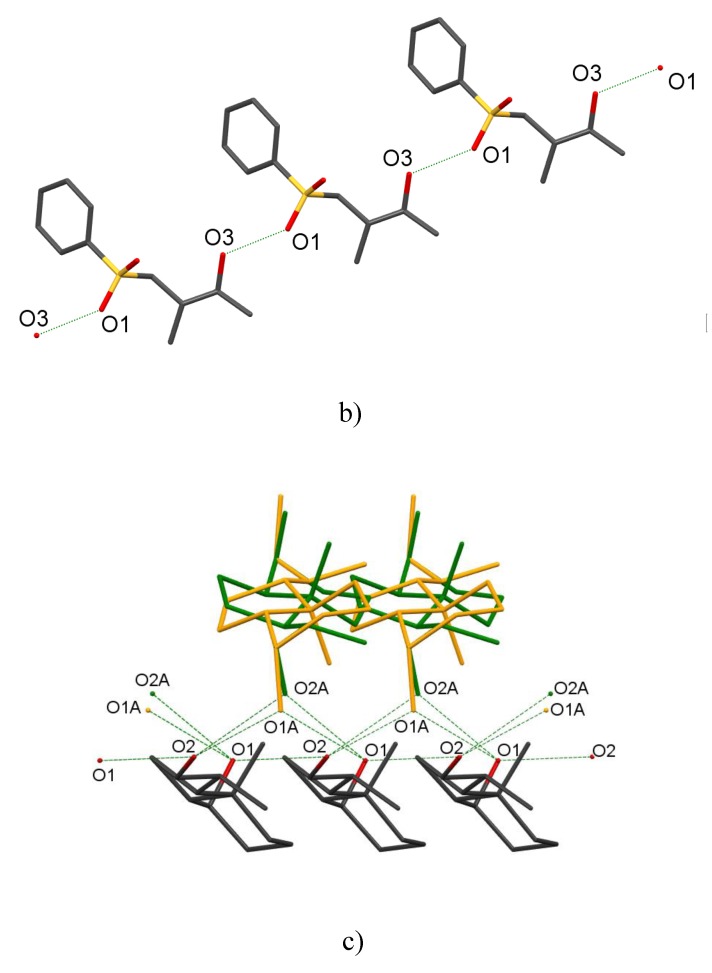
Infinite hydrogen bond motifs in the crystal structures of (**a**) **1**, (**b**) **2**, and (**c**) **3**. The hydrogen atoms were omitted for clarity.

**Figure 5:**
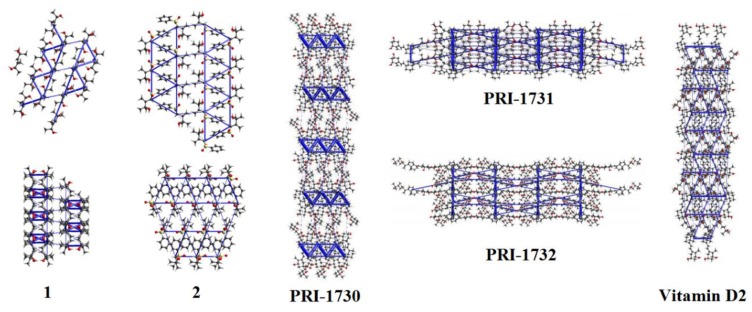
Energy frameworks for **1**, **2**, and vitamin D analogues. Results for all frameworks were presented for the total energy, using the scale factor equal to 50, and the value of energy threshold was equal to 5 kJ/mol.

An infinite chain hydrogen bond motif is essential for interactions of the vitamin D analogues and can also be found in structures **1**, **2**, and **3** (Figure 4). In the next step, we calculated the energy frameworks for **1** and **2** as disorder in **3** limited a possible analysis (Table 3). As expected, the energy framework patterns found for **1** and **2** were in accordance with the pattern found for the vitamin D analogues. These patterns contained infinite chains within all the Coulomb, dispersion, and total energy frameworks. The infinite chains typically form triangular shapes (Figure 5, Figures S5 and S6). The values of interaction energy between the molecules are discussed further below.

#### 2.4.2. Hydrogen Bonds in the Intermediates

**Figure 6:**
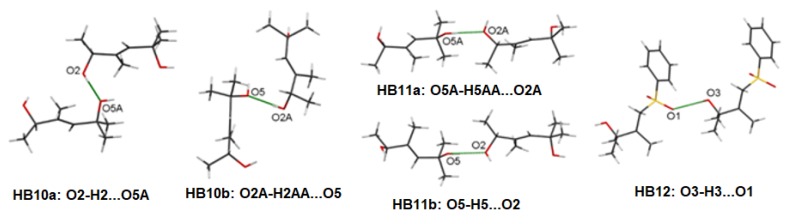
Selected hydrogen bond dimers found in precursors of the vitamin D analogue side chains. The O2 and O5 atoms belong to molecules with (2*R*) absolute configuration. Atoms O2A and O5A belong to molecules with (2*S*) absolute configuration.

Owing to the relationship between hydrogen bond motifs and energy frameworks, we performed further analysis to verify the presence of HB1–HB9 (Figure S7) dimers in compounds **1** and **2**. However, first we would like to clarify the names of hydrogen bonds in these compounds. Hydrogen bonds of **1** are those formed between the 2- and 5-hydroxyls. We marked as HB10 hydrogen bonds where 5-hydroxyls donate their lone electron pairs. Hydrogen bonds with 2-hydroxyl as donors were marked as HB11. As the structure of **1** is racemic, we additionally added *classes a and b*. Dimers where the acceptor is a part of the molecule with absolute configuration (2*R*) were marked as *a*, but *b* when this configuration was (2*S*). For example, dimer O2A-H2AA…O5 is HB10b because the 2-hydroxyl is an acceptor of the hydrogen bond (HB10) and belongs to the molecule with the S absolute configuration (class *b*) (Figure 6).

#### 2.4.3. Hydrogen Bonds in the Intermediates and Vitamin D Analogues

Geometrical analysis showed that the values of the hydrogen bond lengths in **1** are close to the respective values in the following dimers: HB2, HB3, HB6, and HB8 (Figure S7) found in the PRI-1730, PRI-1731, PRI-1732 analogues, and vitamin 1,25D_2_, respectively [16]. Interestingly, we found differences between the distances of identical dimers in different compounds, for example, HB2 had a bond length of 1.921(2)Å and 1.96(3)Å for PRI-1731 and vitamin 1,25D_2_, respectively. The differences between the vitamin D analogues were observed in the positions of the 19-methylene groups in the natural positions for vitamin D_2_, that is, the 19-methylene group attached to the C10 carbon ((5*Z*,7*E*) geometry of the triene system), to modified one, that is, with the methylene group attached to the C4 carbon ((5*E*,7*E*) geometry) in PRI-1731. Therefore, this observation showed that position of the 19-methylene group has a direct influence on the length of the hydrogen bonds. Moreover, our analysis revealed that reported values of HB2 were close to the lengths of HB11a (1.916(2)Å) and HB10a (1.968(1)Å), respectively. This similarity may result from the changes in the configuration of the atoms in the molecules. Hydrogen bond HB11a is formed between two molecules with an (2*S*) configuration, while HB10a is formed between two molecules with the opposite (2*R*) configuration. For HB2, the interacting C1 carbon atom has an *S* configuration in the vitamin D_2_ and *R* in the PRI-1731. Our analysis showed that the geometry of hydrogen bonds in basic intermediates depended on the absolute configuration of interacting carbons. These observations suggest that the effect on the hydrogen bond geometry is influenced by the 19-methylene group. For instance, the geometry of the hydrogen bonds formed between different molecules of **1** hewed closely to the expected parameters for the geometry of hydrogen bonds of vitamin D with a methylene group attached to the C-10. Likewise, the geometry of hydrogen bonds formed between identical molecules of **1** was related to the geometry of hydrogen bonds of vitamin D without methylene group attached to the C-10.

The above relations were further investigated by our analysis of the other dimers. The values for the HB11a bond length were close to that of HB3 of PRI-1731 and PRI-1732. Both of these compounds have methylene groups attached to the C-4. Further, the values of HB3 bond for PRI-1730 with the methylene group attached to the C-10 were close to the value of the HB10b, which was formed between two different compounds. Importantly, C-3 is involved in HB3 and its configuration is (*R*) for the analogues with a methylene group attached to C-10, and (*S*) for the others. As these configurations are opposite to the C-1 atom, this shows that similarity is connected not only to the absolute configuration of the atoms involved, but also to the position of the 19-methylene group.

The next example is HB11b, formed between identical molecules, which has a bond length close to the length of HB6 of PRI-1732. Interestingly, the bond length of HB10b is also similar to that of HB8 of PRI-1732, which is against our relations. However, the HB8 hydrogen bonds are involved in side chain interactions, while this methylene group is not.

In the case of **2**, it appears that HB12 has an interaction distance similar to that of HB7 in the PRI-1732 and HB1 in the PRI-1731 dimers. However, a lack of analogues with an opposite configuration excludes analysis of the influence of configuration of the C8 atom on geometrical similarity. Specifically, the C-8 is not directly connected to the hydroxyl group involved in the hydrogen bond. All these results are summarized in Table 3 and Table 4. Moreover, we performed a search of the Cambridge Structural Databases (CSD), which resulted in two structures (refcodes: MPTSBU20 and MARBIQ), containing both a sulphonyl group forming hydrogen bond and structural fragment corresponding to the end of the side chain in the vitamin D analogues. The lengths of the hydrogen bonds formed in these structures were equal to 2.062(2) Å and 1.984(4) Å, respectively. Interestingly, the hydroxyls involved in the formation of these bonds correspond to 25-hydroxyl for MPTSBU20 and do not correspond to any hydroxyls that occur in the natural vitamins D for MARBIQ. This shows that the configuration of the C8 atom may influence geometrical similarity, as a strong influence of the presence of the sulphonyl group on the formation and geometry of the hydrogen bonds was not found.

#### 2.4.4. Energy Frameworks

Our results clearly showed that the position of 19-methylene group determines the geometry of the hydrogen bonds in the vitamin D analogues. Moreover, the values of the HB lengths are related to the values of the lengths of the hydrogen bonds of the intermediates and depend on their absolute configurations. However, our further analysis (Table 3 and Table 4) shows that there is no simple correlation directly to the energy of these dimers. Geometrical similarity is not simply related to the energetic similarity, as geometrical properties depend on local interactions, whereas energetic properties depend on the interactions of the entire molecule. For instance, the length of HB11a is similar to the lengths of HB2 and HB3, while the energy is similar to the energies of HB3 and HB6. However, for each of the HB10–HB12 dimers, except HB10b, one dimer exists that shows geometrical as well as energetic similarity. These dimers are HB2, HB3, HB6, and HB1 for hydrogen bonds HB10a, HB11a, HB11b, and HB12, respectively.

Although our hypothesis is not fully supported by the results of the interaction energy analysis, this may be explained by the lack of the relevant results for the PRI-1730 structure. Dimers in PRI-1730 form two or three different hydrogen bonds in one dimer including the intramolecular interactions, which affect the final results. Nevertheless, the hypothesis is still supported by the analysis of HB11a and HB11b, which are formed between the same molecules and energetically related to analogues containing the 19-methylene group attached to C-4. These dimers have the same Coulomb and dispersion energy, which are similar to these energies of HB3 and HB6 of PRI-1732, respectively.

## 3. Materials and Methods

### 3.1. Synthesis and Crystallization

Diol **1** and sulfone **2** were synthesized using known methods, whereas Inhoffen–Lythgoe diol **3** was obtained from commercial sources. The crystals of vitamin D precursors **1**, **2**, and **3** were obtained by isotermic crystallization. Analysed compounds were dissolved in the ethyl acetate and crystallized by slow evaporation at room temperature.

### 3.2. Single Crystal X-ray Studies

The CuKα X-ray diffraction data for all crystals were collected using a Supernova Dual Agilent (Rigaku, Wrocław, Poland) 4-circle X-ray diffractometer equipped with an Atlas detector (Rigaku, Wrocław, Poland). The instrument was run with Xcalibur software and was equipped with an CuKα X-ray tube (λ = 1.54184 Å, 50.0 kV, 0.8 mA). The measurements were carried out at 100 K using the Oxford Cryostream cooling device. All crystals were positioned 73 mm from the CCD camera. All frames were measured at 1° intervals. In total, 4782 frames with a counting time of 5–27.5 s, 2771 frames with a counting time of 2.5–15 s, and 2928 frames with a counting time 10–24.7 s were collected for **1**, **2**, and **3** respectively.

Data reduction and analysis were carried out with CrysAlis software [38]. The structures were solved using the Olex2 [39] package and the ShelXS [40] structure solution program using direct methods and refined with the ShelXL [41] package using the least squares minimization.

Refinements were based on F^2^ for all reflections except those with negative intensities. Weighted R factors (*w*R) and all goodness-of-fit (GooF) values were based on F^2^. Conventional R factors were based on the amplitudes, with F set to zero for negative F^2^. The F_o_^2^ > 2σ(F_o_^2^) criterion was applied only for R factor calculations and was irrelevant to the choice of reflections for the refinement. The R factors based on F^2^ were about twice as large as those based on F for all structures. Scattering factors were taken from the International Crystallographic Tables Vol. C [42]. Crystal data, data collection, and refinement details for all compounds are presented in Table 5.

### 3.3. Dimer Calculations

Calculations of energy frameworks [43] were performed using Crystal Explorer 17.5 [44]. Energy frameworks enabled calculations of energy interactions between the whole molecule in the cluster of the nearest neighbour molecules. The CrystalExplorer method is based on the PIXEL method [45], which is a semi-empirical technique for evaluating intermolecular interactions based on integration over the calculated electron densities of single molecules. The monomer wavefunction was used to obtain accurate values of the electrostatic, polarization, and repulsion energies, along with Grimme’s D2 dispersion corrections. Hydrogen bonds were extended to the mean neutron values using LSDB program [46,47]. Owing to presence of disordered regions in structure **3**, calculations were performed only for structures **1** and **2**. Density Functional Theory (DFT) calculation at the B3LYP level of theory and a 6–31 G(d,p) basis set was applied. The level of theory and basis set are in accordance with the previously performed energy frameworks calculations for vitamin D analogues, which are used for a comparison in this study [16]. The results for all frameworks were presented using the scale factor equal to 50 and the value of energy threshold equal to −5 kJ/mol.

### 3.4. CSD Search

Our search of the Cambridge Structural Databases (version 5.37 [19]) resulted in two structures (refcodes: MARBIQ [48] and MPTSBU20[49]), containing both a sulphonyl group forming hydrogen bond with a hydroxyl group and a structural fragment corresponding to the side chain, that is, C24, C25, C26, C27, and O25 atoms including attached hydrogen atoms.

## 4. Conclusions

Vitamins D and their analogues are compounds broadly used as pharmaceutical substances. Broad applications result from interactions with VDR that occur in most cells of the human body. Therefore, vitamins D regulate *i.a.* immune, respiratory, and muscle systems. Vitamin D analogues, such as tacalcitol and calcipotriol, are used against various afflictions such as psoriasis. In this paper, we proved that solving the solid-state structure of vitamin D structural parts as simple intermediates might give a valuable insight into the structure of the final analogue, thus allowing to predict the biological activity of analogues that resist crystallization. We reported three new structures of precursors of vitamin D analogues. These compounds were the building blocks of the side chain (**1** and **2**) and the CD-ring (**3**) of vitamin D analogues. Our study showed that intermediates of the vitamin D analogues crystallize in space groups typical for vitamins D. The geometrical parameters of the analysed compounds were related to corresponding geometrical parameters in vitamin D analogues. Moreover, similar intermolecular interactions were present in both intermediates and final vitamin D molecules. This also includes the most important interaction for vitamin D analogues, that is, the infinite hydrogen bond motif. This finding was supported by analysis of the geometry of these interactions as well as energy framework calculations. We showed that there is a correspondence of not only the interaction type, but also the values of the Coulombic, dispersion, and total energies of for the HB1–HB9 dimers, and this was distinctive for the vitamin D and HB10–HB12 dimers found in the vitamin D intermediates. Interestingly, these connections, particularly geometrical ones, were in accordance with the rule that the relationship between the hydrogen bonds depends on the absolute configuration of compounds and the positions of the 19-methylene in the vitamin D analogues. Therefore, this work also illustrates the influence of the position of the 19-methylene group on the hydrogen bond properties.

## Figures and Tables

**Table 1:** Geometry analysis of side chain of analogues of vitamin D and corresponding bonds from **1** and **2**. Bond lengths given in Å.

	PRI-1730	PRI-1731	PRI-1732	1S	1R	2
C25-C26	1.519 (6)	1.523 (4)	1.524 (3)	1.523 (3)	1.523 (3)	1.506 (6)
C25-C27	1.522 (6)	1.523 (4)	1.524 (3)	1.528 (3)	1.529 (3)	1.531 (5)
C25-O25	1.447 (5)	1.436 (3)	1.452 (3)	1.447 (2)	1.445 (2)	1.441 (5)
C25-C24	1.556 (6)	1.549 (4)	1.552 (3)	1.515 (3)	1.520 (3)	1.536 (5)
C24-C28	1.538 (6)	1.530 (4)	1.532 (3)	n/a	n/a	1.530 (5)
C24-C23	1.538 (5)	1.500 (3)	1.537 (2)	n/a	n/a	1.542 (5)

**Table 2:** Geometry analysis of CD-ring system of analogues of vitamin D and corresponding bonds from **3**. Bond lengths given in Å.

	PRI-1730	PRI-1731	PRI-1732	3
C13-C17	1.558 (5)	1.558 (2)	1.561 (2)	1.563 (3)
C13-C18	1.537 (5)	1.529 (3)	1.533 (2)	1.524 (4)
C13-C14	1.563 (5)	1.554 (3)	1.546 (2)	1.549 (4)
C13-C12	1.541 (5)	1.527 (3)	1.536 (2)	1.526 (4)
C22-C20	1.546 (5)	1.503 (3)	1.548 (2)	1.527 (4)
C17-C20	1.552 (5)	1.540 (3)	1.540 (2)	1.538 (4)
C17-C16	1.552 (5)	1.550 (3)	1.563 (2)	1.554 (4)
C20-C21	1.532 (6)	1.526 (3)	1.518 (3)	1.516 (4)
C14-C8	1.509 (5)	1.507 (3)	1.506 (2)	1.534 (4)
C14-C15	1.522 (5)	1.524 (3)	1.516 (2)	1.516 (6)
C8-C9	1.514 (5)	1.510 (3)	1.507 (3)	1.512 (7)
C11-C12	1.526 (5)	1.537 (3)	1.543 (2)	1.543 (4)
C11-C9	1.536 (6)	1.528 (3)	1.533 (3)	1.525 (7)
C16-C15	1.561 (5)	1.545 (3)	1.545 (2)	1.556 (4)

**Table 3:** Results of energy framework calculations for **1** and **2** and lengths of the analysed hydrogen bonds.

Molecule	Hydrogen Bonds		Length of Hydrogen Bonds [Å]	Coulomb Energy [kJ/mol]	Dispersion Energy [kJ/mol]	Total Energy [kJ/mol]
**1**	O2-H2…O5A	HB10a	1.968 (1)	−47.6	−19.7	−35.8
	O5A-H5AA…O2A	HB11a	1.916 (2)	−46.4	−19.5	−43.5
	O2A-H2AA…O5	HB10b	1.937 (2)	−49.2	−27.1	−43.7
	O5-H5…O2	HB11b	1.930 (2)	−46.4	−19.5	−43.5
**2**	O3-H3…O1	HB12	2.011 (3)	−34.1	−14.3	−30.3

**Table 4:** Geometrical and energetic similarities of the hydrogens bonds of **1** and **2** with a comparison to vitamin D analogues.

Molecule	Hydrogen Bonds		Geometrical Similarity	Energetic Similarity		
				Coulomb Energy	Dispersion Energy	Total Energy
**1**	O2-H2…O5A	HB10a	HB2	HB2/HB3	HB6	HB3/HB6
	O5A-H5AA…O2A	HB11a	HB2, HB3	HB3	HB6	-
	O2A-H2AA…O5	HB10b	HB3, HB8	HB2	-	-
	O5-H5…O2	HB11b	HB6	HB3	HB6	-
**2**	O3-H3…O1	HB12	HB7, HB1	HB1	HB3	HB2

**Table 5:** Experimental details of precursors **1**, **2**, and **3**.

Crystal Data			
	1	2	3
Chemical formula	C_8_H_16_O_2_	C_12_H_18_O_3_S	C_9.75_H_17.75_O_1.5_
*M*_r_	144.21	242.32	158.99
Crystal system, space group	Monoclinic, *P*2_1_/*c*	Orthorhombic, *P*2_1_2_1_2_1_	Monoclinic, C2
Temperature (K)	100	100	100
*a*, *b*, *c* (Å)	20.4858 (5), 6.02000 (12), 15.3758 (4)	5.63736 (9), 7.88326 (9), 28.8351 (4)	18.59 (3), 6.891 (7), 15.65 (2)
β (°)	111.124 (3)		107.53 (16)
*V* (Å^3^)	1768.80 (7)	1281.45 (3)	1912 (5)
*Z*	8	4	8
Radiation type	Cu *K*α		
µ (mm^−1^)	0.61	2.18	0.56
Crystal size (mm)	0.39 × 0.1 × 0.09	0.27 × 0.22 × 0.15	0.23 × 0.07 × 0.06
**Data Collection**			
Diffractometer	SuperNova, Dual, Cu at zero, Atlas		
Absorption correction	Multi-scan *CrysAlis PRO* 1.171.38.41 (Rigaku Oxford Diffraction, 2015) Empirical absorption correction using spherical harmonics, implemented in SCALE3 ABSPACK scaling algorithm.		
*T*_min_, *T*_max_	0.784, 1.000	0.775, 1.000	0.808, 1.000
No. of measured, independent and observed [I>2σ(I)] reflections	13,249, 3712, 3654	12,735, 2669, 2636	19,999, 3648, 3425
*R*_int_	0.028	0.027	0.033
(sin θ/λ)_max_ (Å^−1^)	0.632	0.631	0.617
**Refinement**			
*R*[*F*^2^ > 2σ(*F*^2^)], *wR*(*F*^2^), *S*	0.066, 0.162, 1.15	0.045, 0.107, 1.10	0.0427, 0.109, 1.07
No. of reflections	3712	2669	3684
No. of parameters	193	149	343
No. of restraints	0	0	23
H-atom treatment	H-atom parameters constrained		H-atoms treated by a mixture of independent and constrained refinement
Δρ_max_, Δρ_min_ (e Å^−3^)	0.32, −0.19	0.43, −0.53	0.18, −0.23
Absolute structure	n/a	Flack x determined using 1024 quotients [(I+)−(I−)]/[(I+)+(I−)] (Parsons, Flack and Wagner, Acta Cryst. B69 (2013) 249–259).	Absolute structure: Flack x determined using 1428 quotients [(I+)−(I−)]/[(I+)+(I−)] (Parsons, Flack and Wagner, Acta Cryst. B69 (2013) 249–259).
Absolute structure parameter	n/a	0.011 (8)	−0.06 (8)

Computer programs: *CrysAlis PRO* 1.171.38.41 (Rigaku OD, 2015), *SHELXS* (Sheldrick, 2008), *SHELXL* (Sheldrick, 2015), Olex2 (Dolomanov et al., 2009).

## Supplementary Materials

The following are available online. Figure S1: Packing of the crystal lattice of precursor 1, viewed along a-, b-, and c-directions. The H-atoms were omitted for clarity; Figure S2: Packing of the crystal lattice of precursor 2, viewed along a-, b-, and c-directions. The H-atoms were omitted for clarity; Figure S3: Packing of the crystal lattice of intermediate 3, viewed along a-, b-, and c-directions. The H-atoms were omitted for clarity; Figure S4: Structure of 3: (a) part 0 as well as the disordered part –1 and (b) disordered part –1 with hydrogen atoms. Atoms marked as green and orange have occupancy equal to 0.5. Atoms marked by grey and red, as well as atoms connected to both green and orange atoms, have occupancy equal to 1; Figure S5: Energy frameworks of precursor 1: Coulomb energy (red), dispersion energy (green), and total energy (blue) viewed along a-, b-, and c-directions; Figure S6: Energy frameworks of precursor 2: Coulomb energy (red), dispersion energy (green), and total energy (blue) viewed along a-, b-, and c-directions; Figure S7: HB1–HB9 hydrogen bond dimers found in vitamin D analogues; Cif and checkcif files for **1**, **2**, and **3**; 3D figure of **3** with H-atoms, 3D figure of **3** with H-atoms omitted.
