# Supplementary material for: Relation between Crystal Structures of Precursors and Final Products: Example of Vitamin D Intermediates
Source: Molecules. 2020 Apr 14;25(8):1802. doi: 10.3390/molecules25081802 (PMC7221991; doi:10.3390/molecules25081802)
Supplement: Supplementary file 1 [file molecules-25-01802-s001.pdf]

molecules

# Relation between crystal structures of precursors and final products: example of vitamin D intermediates

**Monika Wanat[1,2], Maura Malinska[2], Andrzej Kutner[3] and Krzysztof Wozniak[4,*]**

1 Biological and Chemical Research Centre, Department of Chemistry, University of Warsaw, 101 Żwirki i Wigury, Warszawa, 02-089, Poland
2 College of Inter-Faculty Individual Studies in Mathematics and Natural Sciences (MISMaP), University of Warsaw, 2C Stefana Banacha, Warszawa, 02-097, Poland
3 Faculty of Pharmacy, Department of Bioanalysis and Drug Analysis, Medical University of Warsaw, 1 Stefana Banacha, Warszawa, 02-097, Poland

## Supporting information

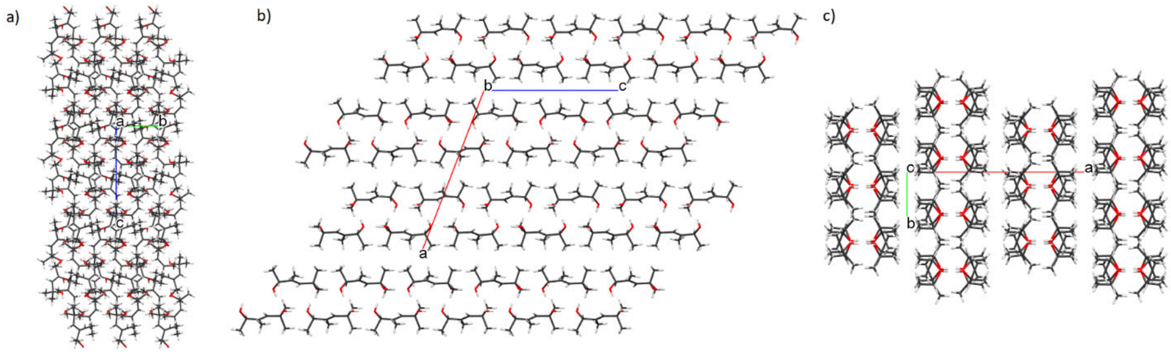


**Figure S1** Packing of the crystal lattice of precursor **1**, viewed along a, b and c-directions. The H-atoms were omitted for clarity.

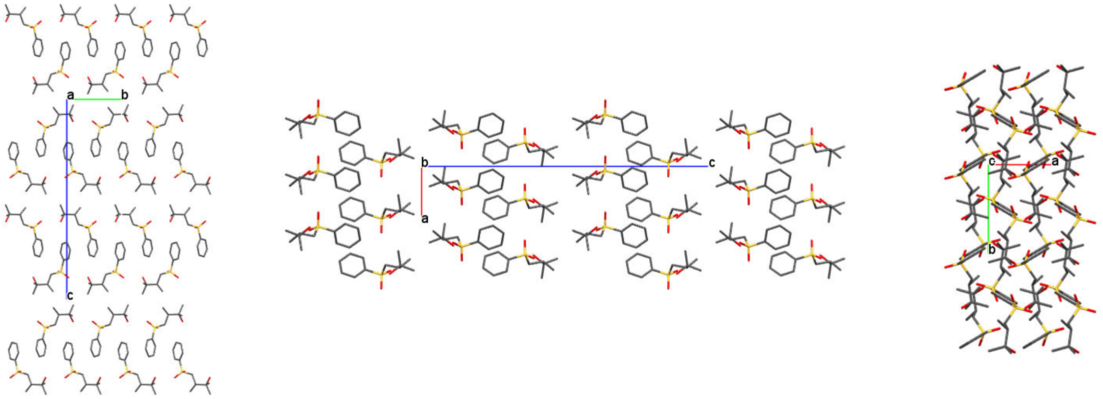


**Figure S2** Packing of the crystal lattice of precursor **2**, viewed along a, b and c-directions. The H-atoms were omitted for clarity.

molecules

MDPI

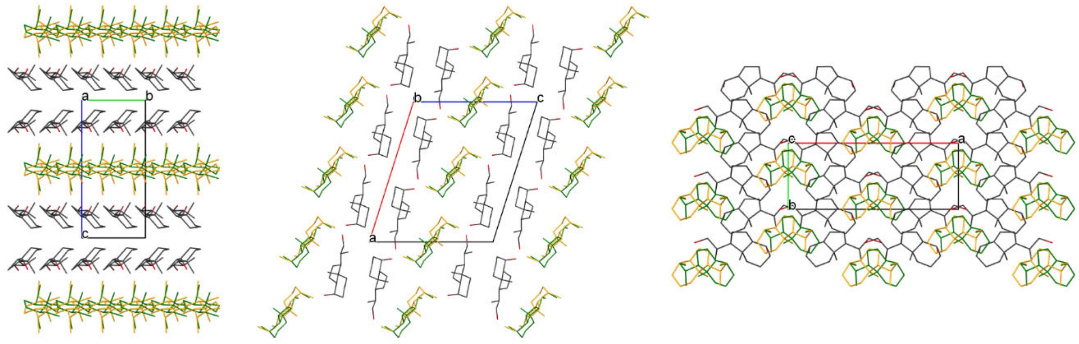

**Figure S3** Packing of the crystal lattice of intermediate **3**, viewed along a, b and c-directions. The H-atoms were omitted for clarity.

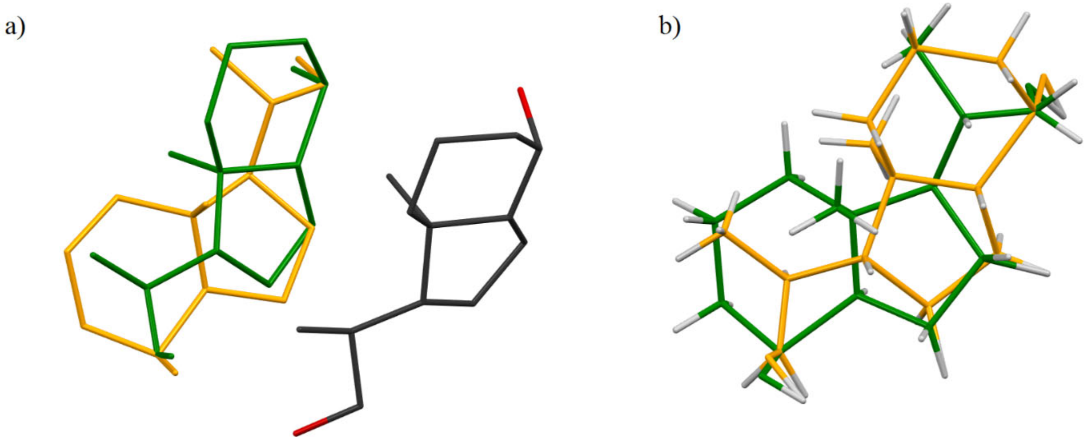


**Figure S4** Structure of **3**: a) part 0 as well as the disordered part -1 and b) disordered part -1 with hydrogen atoms. Atoms marked as green and orange have occupancy equal to 0.5. Atoms marked by grey and red as well as atoms connected to both, green and orange atoms, have occupancy equal to 1.

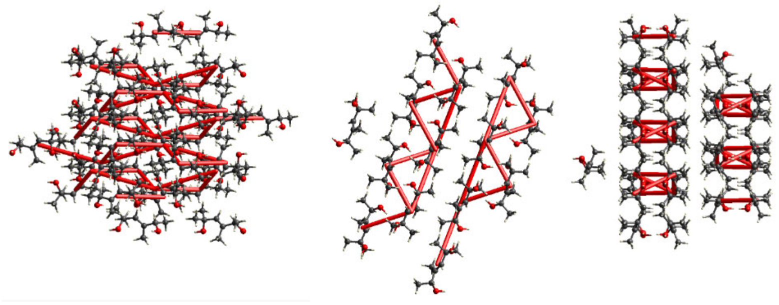

*molecules*

MDPI

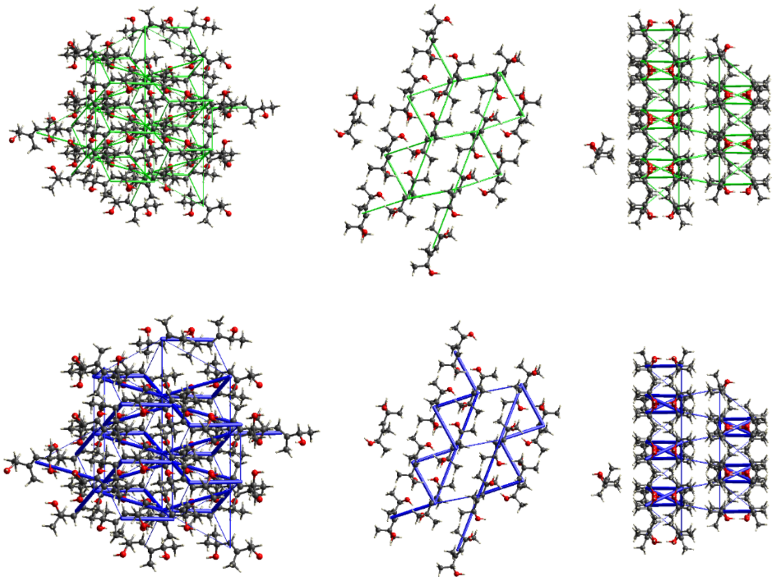

**Figure S5** Energy frameworks of precursor **1**: Coulomb energy (red), dispersion energy (green) and total energy (blue) viewed along a, b and c-directions.

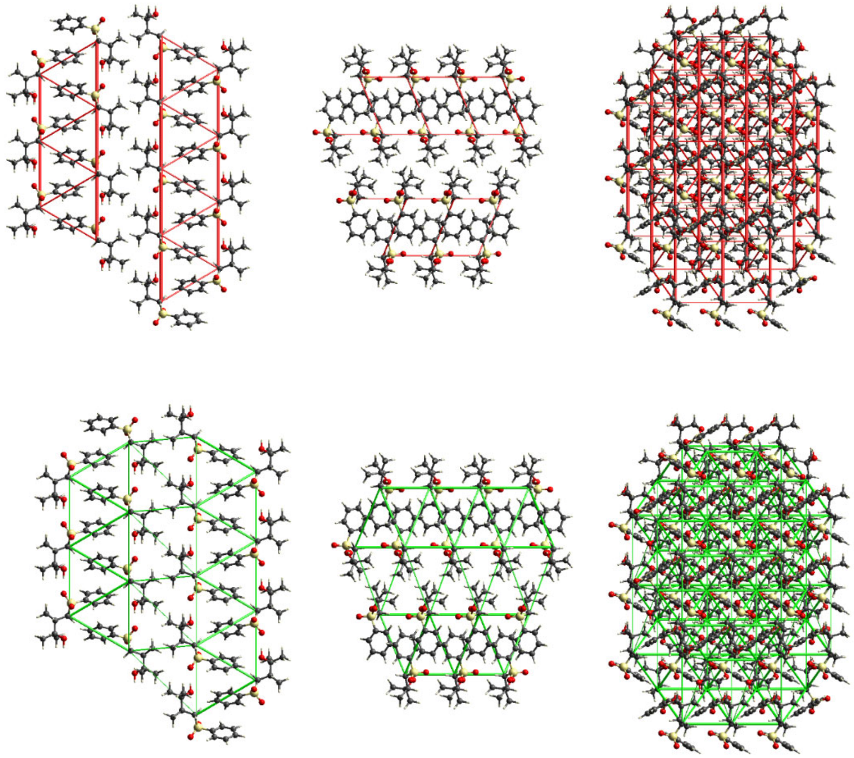

molecules
MDPI

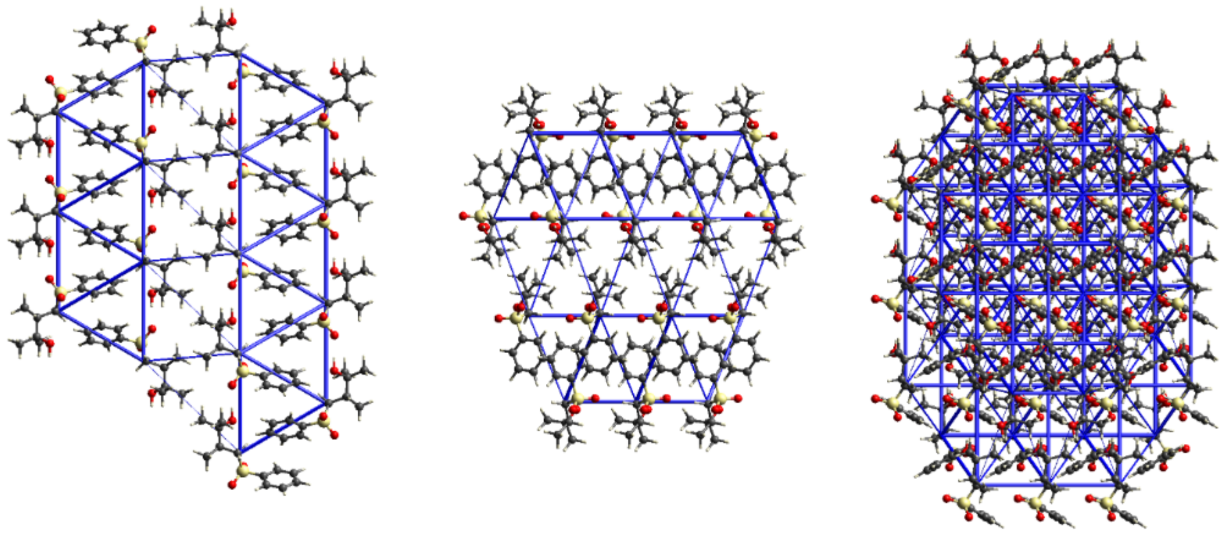

**Figure S6** Energy frameworks of precursor **2**: Coulomb energy (red), dispersion energy (green) and total energy (blue) viewed along a, b and c-directions.

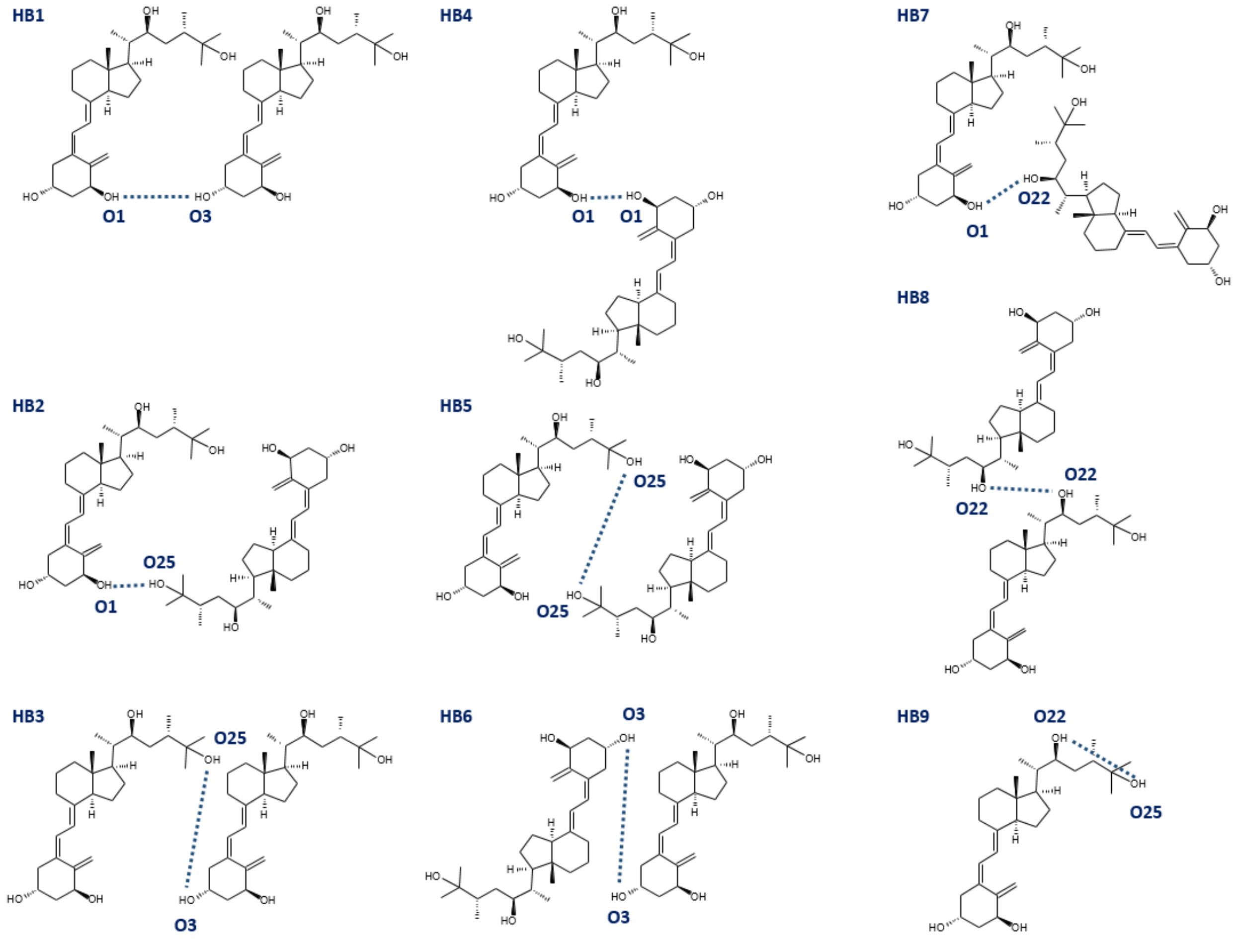


**Figure S7** HB1-HB9 hydrogen bond dimers found in vitamin D analogues.
